# Predicting metastasis in gastric cancer patients: machine learning-based approaches

**DOI:** 10.1038/s41598-023-31272-w

**Published:** 2023-03-13

**Authors:** Atefeh Talebi, Carlos A. Celis-Morales, Nasrin Borumandnia, Somayeh Abbasi, Mohamad Amin Pourhoseingholi, Abolfazl Akbari, Javad Yousefi

**Affiliations:** 1 Colorectal Research Center, Iran University of Medical Sciences, Tehran, Iran; 2 British Heart Foundation Cardiovascular Research Centre, University of Glasgow, Glasgow, UK; 3 Institute of Cardiovascular and Medical Sciences, University of Glasgow, Glasgow, UK; 4 Urology and Nephrology Research Center, Shahid Beheshti University of Medical Sciences, Tehran, Iran; 5 Department of Mathematics, Isfahan (Khorasgan) Branch, Islamic Azad University, Isfahan, Iran; 6 Gastroenterology and Liver Diseases Research Center, Research Institute for Gastroenterology and Liver Diseases, Shahid Beheshti University of Medical Sciences, Tehran, Iran; 7 Colorectal Research Center, Iran University of Medical Sciences, Tehran, Iran; 8 Department of Internal Medicine, School of Medicine, Iran University of Medical Sciences, Tehran, Iran

**Keywords:** Cancer, Mathematics and computing

## Abstract

Gastric cancer (GC), with a 5-year survival rate of less than 40%, is known as the fourth principal reason of cancer-related mortality over the world. This study aims to develop predictive models using different machine learning (ML) classifiers based on both demographic and clinical variables to predict metastasis status of patients with GC. The data applied in this study including 733 of GC patients, divided into a train and test groups at a ratio of 8:2, diagnosed at Taleghani tertiary hospital. In order to predict metastasis in GC, ML-based algorithms, including Naive Bayes (NB), Random Forest (RF), Support Vector Machine (SVM), Neural Network (NN), Decision Tree (RT) and Logistic Regression (LR), with 5-fold cross validation were performed. To assess the model performance, F1 score, precision, sensitivity, specificity, area under the curve (AUC) of receiver operating characteristic (ROC) curve and precision-recall AUC (PR-AUC) were obtained. 262 (36%) experienced metastasis among 733 patients with GC. Although all models have optimal performance, the indices of SVM model seems to be more appropiate (training set: AUC: 0.94, Sensitivity: 0.94; testing set: AUC: 0.85, Sensitivity: 0.92). Then, NN has the higher AUC among ML approaches (training set: AUC: 0.98; testing set: AUC: 0.86). The RF of ML-based models, which determine size of tumor and age as two essential variables, is considered as the third efficient model, because of higher specificity and AUC (84% and 87%). Based on the demographic and clinical characteristics, ML approaches can predict the metastasis status in GC patients. According to AUC, sensitivity and specificity in both SVM and NN can be regarded as better algorithms among 6 applied ML-based methods.

## Introduction

Gastric cancer (GC) is considered as third invasive malignant growth across the globe^1^. Incidence of GC may occur by genetic and environmental effects in developing countries^2^. Although the morbidity and mortality of GC have reduced over the past few decades in some nations, the malignancy has remained the fourth leading cause of cancer-related deaths over the world^3^. The mortality of the cancer is also growing and it endangers people's health among Iranian society^4^.

Various automated computational processes enable machines to analysis data. Machine learning (ML) is a branch of artificial intelligence that serves a series of algorithms from training data. ML algorithms identify patterns in data and associate the patterns with distinct classes of records to predict a closing situation. Recently, according to complexity and hugeness of medical data, scientists can apply ML to predict disease risk^5,6^. Moreover, the methods have critical application value in assisting disease diagnosis and predicting clinical outcomes^7^. Compared with common regression models, ML approaches are identified by their superior performance in predicting results within large data bases^8^.

Moreover, a various number of researchers are using several types of MLs in medical sciences domains, such as pancreatic, colorectal, lung cancers^1,9,10^. The importance of ML models are given in a few GC studies. These approaches were evaluated to predict the lymph node metastasis in GC patients^11–17^. Arai et al. (2022) suggested ML method to predict the risk of GC in patients by information on gastric atrophy and intestinal metaplasia at the initial EGD^11^. Fan et al. used ML-based approaches to predict of lymphovascular invasion status (LVI), which was related to metastasis and poor survival in GC patients^12^. Also, an advanced model was expanded to comprise radiomics clinical and features attributes to boost the performance of the model. Some studies also have advised ML methods to predict metastasis in GC patients^13–17^. Yang et al. (2022) obtained some factors that depend on lymph node metastasis in GC patients. Then, they constructed 5 algorithms of ML in a retrospective dataset^15^. All prediction models of their study demonstrated accuracy between 70 and 81%. Zhou et al. (2021) presented ML algorithm on lymph node metastasis (LNM) in patients who suffered from poorly differentiated-type intramucosal GC^17^. Among the seven algorithm models, the highest and the lowest accuracy rate were related to Gradient Boosting (0.95) and LR (0.63), respectively.

In the study, we aimed to predict the metastasis status on GC patients using ML-based models, including decision tree (DT), random forest (RF), Naive Bayes (NB), Support Vector Machine (SVM), Neural Network (NN) and Logistic Regression (LR). Model performance was evaluated using precision, F1 score, sensitivity, specificity, area under the curve (AUC) of recevier oparating characteristic (ROC) curve and precision-recall AUC (PR-AUC).

## Material and methods

The historical cohort study included 733 patients who were diagnosed as having GC at Taleghani tertiary Hospital. The ML-based classifiers were evaluated to predict metastais status in patients with GC. The inclusion criteria were definitive pathological diagnosis of primary gastric adenocarcinoma and tumor metastasis from 2014 to 2019. Moreover, GC patients who their overall or relative survival after metastasis was assessable based on clinical data, were included in the study. Also, the exclusion criteria included the patients without examination, unavailable or incompleted clinical data. The another criterion was low image quality or small tumor region hard to identify on CT images.

The outcome variable was metastasis, the development of secondary malignant growths at a distance from a primary site of cancer. Also, demographical and clinical variables were considered as predictive factors, including age, sex, marital status, body mass index (BMI), history of smoking, family history, tumor size, grade of tumor, treatment types, the number of involved lymph nodes. First, the univariable statistical analysis was performed to identify the significant factors related to metastasis. Then ML-based algorithms including NB, RF, SVM, NN, DT and LR were implemented to create models for prediction of metastasis in GC patients. This survey was compliant with the principles of the Helsinki Declaration. The methods were performed in accordance with the relevant guidelines and regulations. The present study was approved by the Ethics Committee at Iran University of Medical Sciences (IR.IUMS.REC. 1401-3-49-23823). The need for informed consent has been waived by ethics committee.

### Data preprocessing

At the data preprocessing stage, we synthesized statistically missing values. To impute of missing data, model based imputer approaches were applied. The dataset used in this study, consisting of 733 cases with 10 features, described each patient’s demographical and clinical characteristics, as well as metastasis status as an outcome collected by electronic records. Features with more categorical values were transformed into discrete values using binning discretization mechanisms. Continuizes categorical variables (with one-hot-encoding) and normalizes the data by centering to mean and scaling to standard deviation of 1, were also performed. For NB modelling, numeric values were categorized to four binaries with equal frequency. In our data, the metastasis rate distribution was inherently not balanced (35:65), which seems to be slightly imbalance. Therefore, over-sampling methods were adopted to balance the data. Finally, entire dataset was split by 5-fold cross validation method in the approximate ratio of 4:1 to the training and testing sets.

### Data balancing

The metastasis rate distribution was considered slightly imbalanced in the dataset (with 35 metastasis vs 65 non- metastasis ratios). Therefore, the oversampling technique was applied. In this way, the more cases from minority class are added to balance the data in both classes. Based on this method, new records of metastasis were added, and a new dataset was created. The different ML algorithms were implemented on the original data and balanced data and the results were compared.

### Model development

ML models were developed on selected variables using 5-fold validation method. In this way the data were randomly split into 5-fold. The models were built on the 4 folds and one-fold is left to test the models. In other worlds, data were randomly split into 80% for the training and 20% for the testing each time. Afterwards, NB, RF, SVM, NN, DT and LR were implemented to make models for prediction of metastasis in GC patients.

Hyperparameters was manually selected based on experience to have a baseline result to enable subsequent comparisons. Models were trained with the selected hyperparameters, and scored on the validation data. This process was repeated until the results were satisfiable.

### Neural network

NN approach is similar to natural neural networks and recognize the relationship between variables in a data set through hidden layers and nodes^18^. Considering the various type of NN, the selection of the algorithm was performed by trial and error in the present study. The multi-layer perceptron network was applied. Activation function of the rectified linear unit function and weight optimization of stochastic gradient-based optimizer were used in this modelling.

### Decision tree and random forest

Decision tree, as a non-parametric supervised learning algorithms, splits the data into nodes and construct hierarchical graph, with a structure like trees, which consist branches, root node, leaf nods and internal nodes^19^. The algorithm produces a chart, which has an understandable representation. We used a DT with forward pruning, which split data based on class purity. The RF constructs a set of decision trees by bootstrap sampling from the training data.

Whereas the Gini index is commonly performed as the splitting criterion in classification trees, the corresponding impurity importance is often called Gini importance. The index is so popular because it is fast and simple to calculate^20^. The impurity importance is recognized to be biased in favor of variables with many possible split points, including categorical variables with many categories or continuous variables^21^.

### Support vector machine

The SVM algorithms map the input features to a new higher dimensional space. The hyperplane maximizes the margin between the classes^22^. A Radial basis function kernel was run in this study. The cost 1.00, regression lost 0.1, and numerical tolerance 0.001 was set in the modeling.

### Nave Bayes

Nave Bayes algorithm is a probabilistic classifier which is based on Bayes theorem. This method is fast and has robust performance.

**Figure 1:**
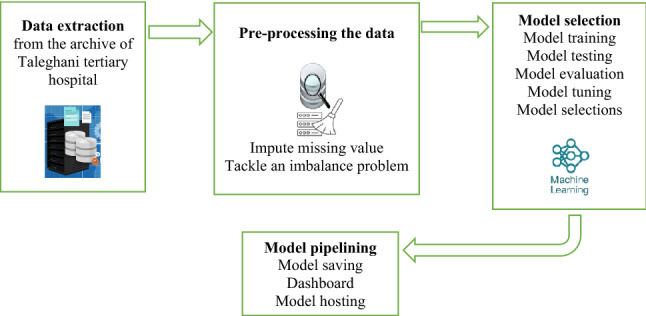
Framework of methodology.

Figure 1 reveals the flowchart of preprocessing of the data and model selection.

### Statistical analysis

The descriptive analyses were applied by the mean ± SD for quantitative and frequency (percentage) for qualitative variables. We did the chi-square test to evaluate the relationship between two categorical factors. Moreover, independent sample T-test was performed to compare means between two groups. Then, ML-based models, including NB, RF, SVM, NN, DT and LR were applied to predict metastasis in patients with GC. Afterwards, the k-fold cross-validation method was performed to validate the model. The value of K was considered equal to 5. Finally, precision, sensitivity, specificity, AUC of ROC curve, F1 score, and precision-recall AUC (PR-AUC) were calculated in original and balanced datasets as well as separately for train and test subsets. The Orange3 version 3.21.0 was used to perform oversampling technique and ML algoritms. The SPSS 23 software was performed to univariable statistical analyses. The R-Studio version 4.2.0 was applied to obtain the figures of AUC-PR. The two-sided p-values smaller than (α = 0.05) is considered as statistically significantly.

### Ethics approval and informed consent

The study was conducted according to the guidelines of the Declaration of Helsinki, and approved by the Ethics Committee of Iran University of Medical Sciences (IR.IUMS.REC. 1401-3-49-23823). Informed consent has been waived by the Ethics Committee of Taleghani tertiary hospital.

## Results

We included 733 patients who underwent at least one type of treatments due to GC at the tertiary hospital. Among those population, 262 (35.7%) patients had metastasis. The mean age of patients who experienced metastasis was 60.02 ± 13.18 and it was 59.53 ± 12.56 in patients without metastasis. The number of females were 130 (27.6%) and 81 (30.9%) in both with and without metastasis groups, respectively. Table 1 demonstrates the demographical and clinical features of the GC patients. Also, it revealed that several variables, such as tumor size, age, grade of tumor, the number of involved lymph node, treatment type, BMI, marital status and history of smoking had significant difference between metastasis and non- metastasis patients (*P* < 0.05).

**Table 1:** Baseline characteristics in patients with GC based on metastatic and non-metastatic status.

	Metastasis		*P* value
	No (n = 471)	Yes (n = 262)	
Age, mean (SD)	59.53 (12.56)	60.02 (13.18)	.616
Sex, n (%)			
Female	130 (61.6)	81 (38.4)	.342
Male	341 (65.3)	181 (34.7)	
Marital Status, n (%)			
Married	461 (64.0)	259 (36.0)	.398
Single	10 (76.9)	3 (23.1)	
BMI, n (%)			
Underweight	99 (62.7)	59 (37.3)	.321
Normal	285 (63.6)	163 (36.4)	
Overweight	60 (67.4)	29 (32.6)	
Obese	27 (71.1)	11 (28.9)	
Smoking, n (%)			
Never used	306 (66.2)	156 (33.8)	.145
Previous or current user	165 (60.9)	106 (39.1)	
Family history, n (%)			
No	340 (61.8)	210 (38.2)	.017
Yes	131 (71.6)	52 (28.4)	
Grade of tumor, n (%)			
Poorly	157 (78.5)	43 (21.5)	< .001
Moderately	114 (83.2)	23 (16.8)	
Well	85 (76.6)	26 (23.4)	
Undifferentiated	115 (40.4)	170 (59.6)	
Number of involved lymph node, n (%)			
N1	154 (73.3)	56 (26.7)	.007
N2	251 (60.2)	166 (39.8)	
N3	66 (62.3)	40 (37.7)	
Tumor size, n (%)			
T1	25 (89.3)	3 (10.7)	< .001
T2	68 (93.2)	5 (6.8)	
T3	276 (94.8)	15 (5.2)	
T4	102 (29.9)	239 (70.1)	
Type of treatment, n (%)			
Other treatments	366 (81.9)	81 (18.1)	< .001
Surgery	105 (36.7)	181 (63.3)	

**Figure 2:**
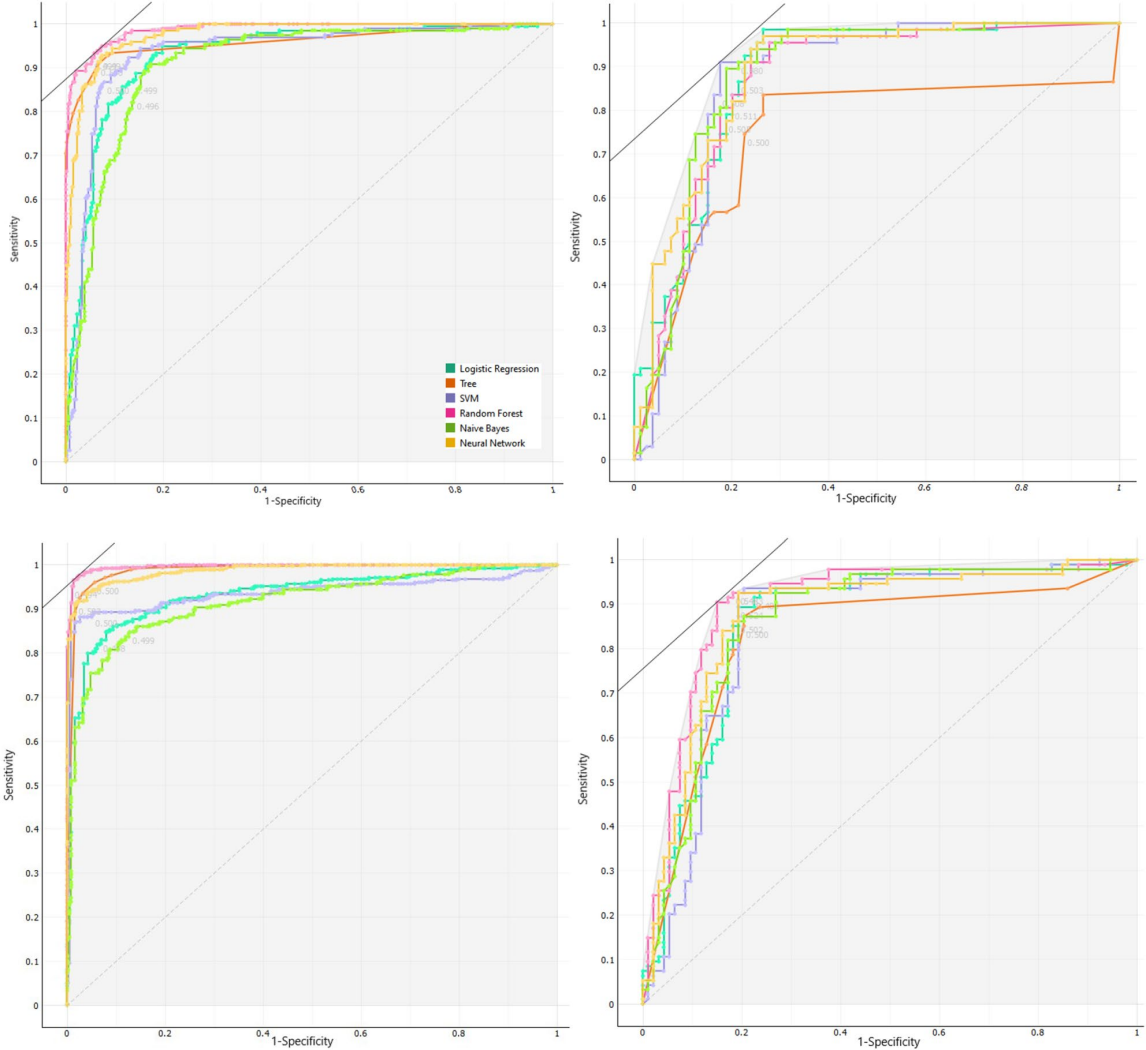
ROC curves for different ML algorithms for predicting metastasis in gastric cancer patients. (Top left: Train original data, Top right: Test original data, Down left: Train balanced data, Down right: Test balanced data).

Then, different approaches of ML were used to predict the metastasis situation in patients with GC. The comparative criteria of models, such as F1 score, precision, sensitivity, specificity and AUC of ROC curve are given in Table 2. The indices, explain the performance of these models in clinical studies, were obtained by 5-fold cross-validation. The sensitivity and specificity of the SVM and NN models were regarded as the best among other models. In addition, RF had AUC 98% and 87% in train and test datasets, sequently. The results of train and test of balanced datasets are similar to original datasets. Therefore, over-sampling methods were confirmed by balance the data. The ROC curve were made to illustrate the performance of a classification model (Fig. 2). The ROC curves were presented in original and balanced data, separately for train and test datasets. The weakest performance was belonged to the DT method in test subset of both original and balance datasets.

**Table 2:** Model performance to predict metastasis in ML approaches using 5-fold cross validation in original data and Balance data with oversampling method.

Models	Dataset		Sensitivity%	Specificity%	Precision%	F1	AUC
LR	Original data	Train	.86	.86	.76	.81	.93
		Test	.86	.78	.77	.81	.88
	Balanced data	Train	.93	.83	.88	.87	.93
		Test	.90	.78	.84	.84	.86
NN	Original data	Train	.93	.93	.88	.88	.98
		Test	.76	.78	.75	.77	.86
	Balanced data	Train	.98	.87	.95	.95	.99
		Test	.91	.81	.86	.86	.87
RF	Original data	Train	.89	.95	.92	.90	.98
		Test	.80	.83	.80	.80	.87
	Balanced data	Train	.98	.95	.96	.96	.99
		Test	.91	.78	.85	.85	.87
NB	Original data	Train	.83	.85	.74	.79	.90
		Test	.89	.78	.77	.83	.88
	Balanced data	Train	.87	.84	.86	.86	.91
		Test	.86	.81	.83	.83	.86
DT	Original data	Train	.82	.97	.94	.88	.96
		Test	.58	.78	.69	.63	.75
	Balanced data	Train	.94	.96	.95	.95	.98
		Test	.80	.81	.80	.80	.83
SVM	Original data	Train	.94	.86	.77	.84	.94
		Test	.92	.76	.76	.85	.85
	Balanced data	Train	.98	.87	.93	.92	.93
		Test	.93	.80	.87	.86	.85

Abrivation: Naive Bayes (NB), Random Forest (RF), Support Vector Machine (SVM), Neural Network (NN), Desicion Tree (RT) and Logistic Regression (LR).

**Figure 3:**
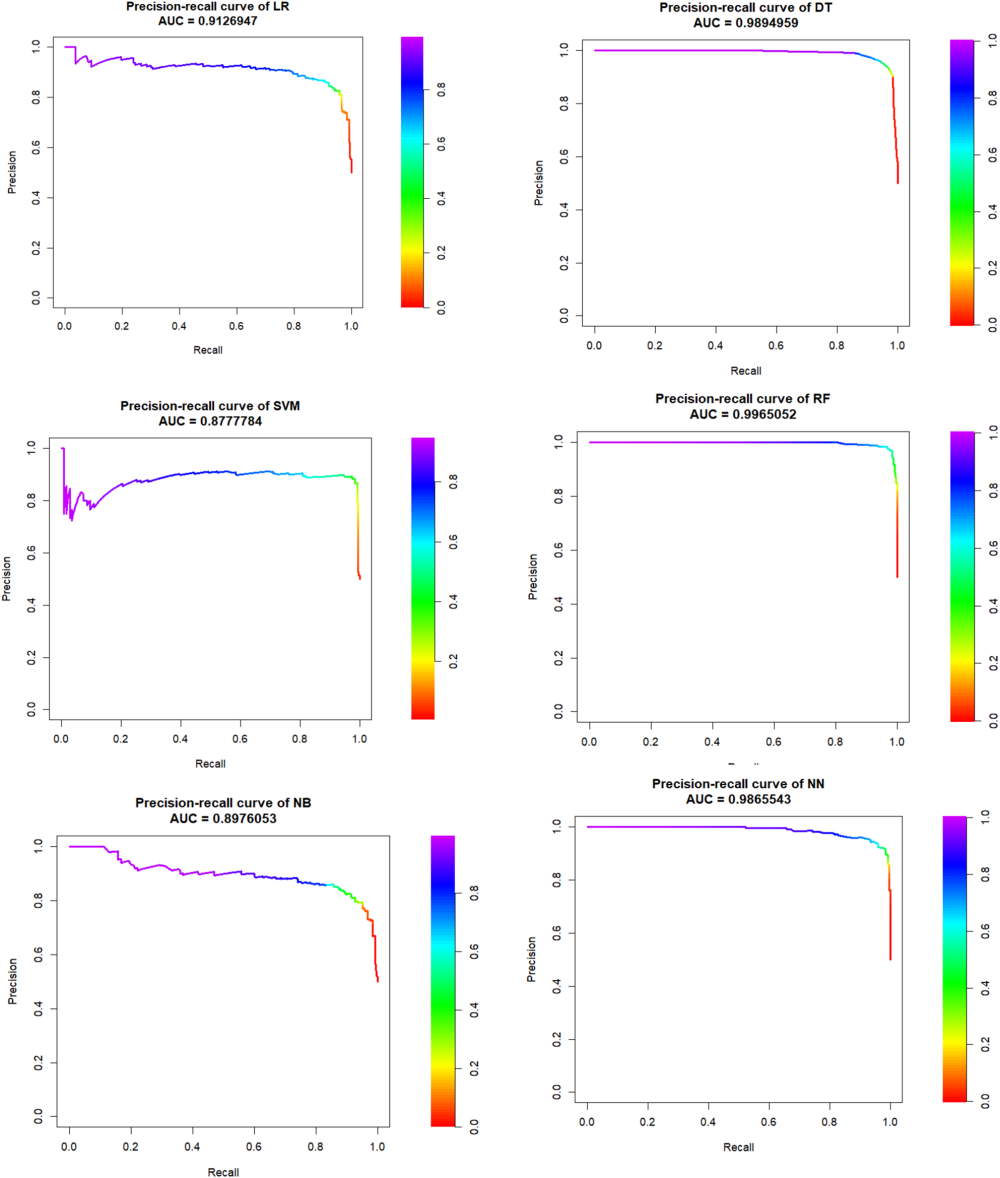
Precision-recall curve and its AUC in six ML algorithms of balanced train dataset of gastric cancer patients.

**Figure 4:**
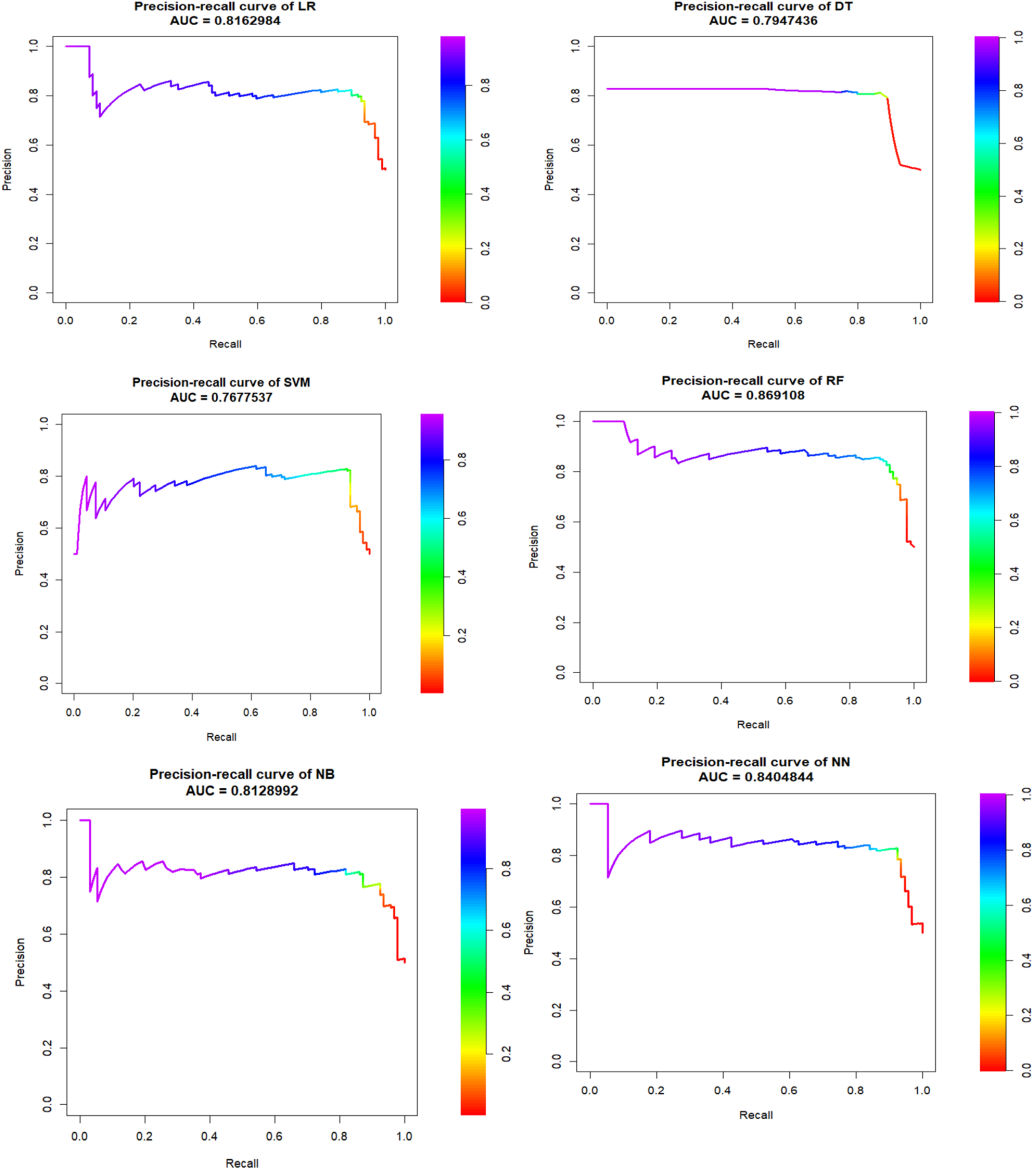
Precision-recall curve and its AUC in six ML algorithms of balanced test dataset of gastric cancer patients.

Figures 3 and 4 is drawn based on PR-AUC in balanced train and test dataset, respectively. They showed all ML-based algorithms have the high PR-AUC in RF, DT and NN (~ 1). The figure demonstrated that though all PRAUC values are more that 0.78, and the PRAUC of SVM, NN and RF seems to be similar to each other. The PRAUC figures of train and test of original datasets are given in Supplementary file, that those figures confirm the balanced datasets.

**Figure 5:**
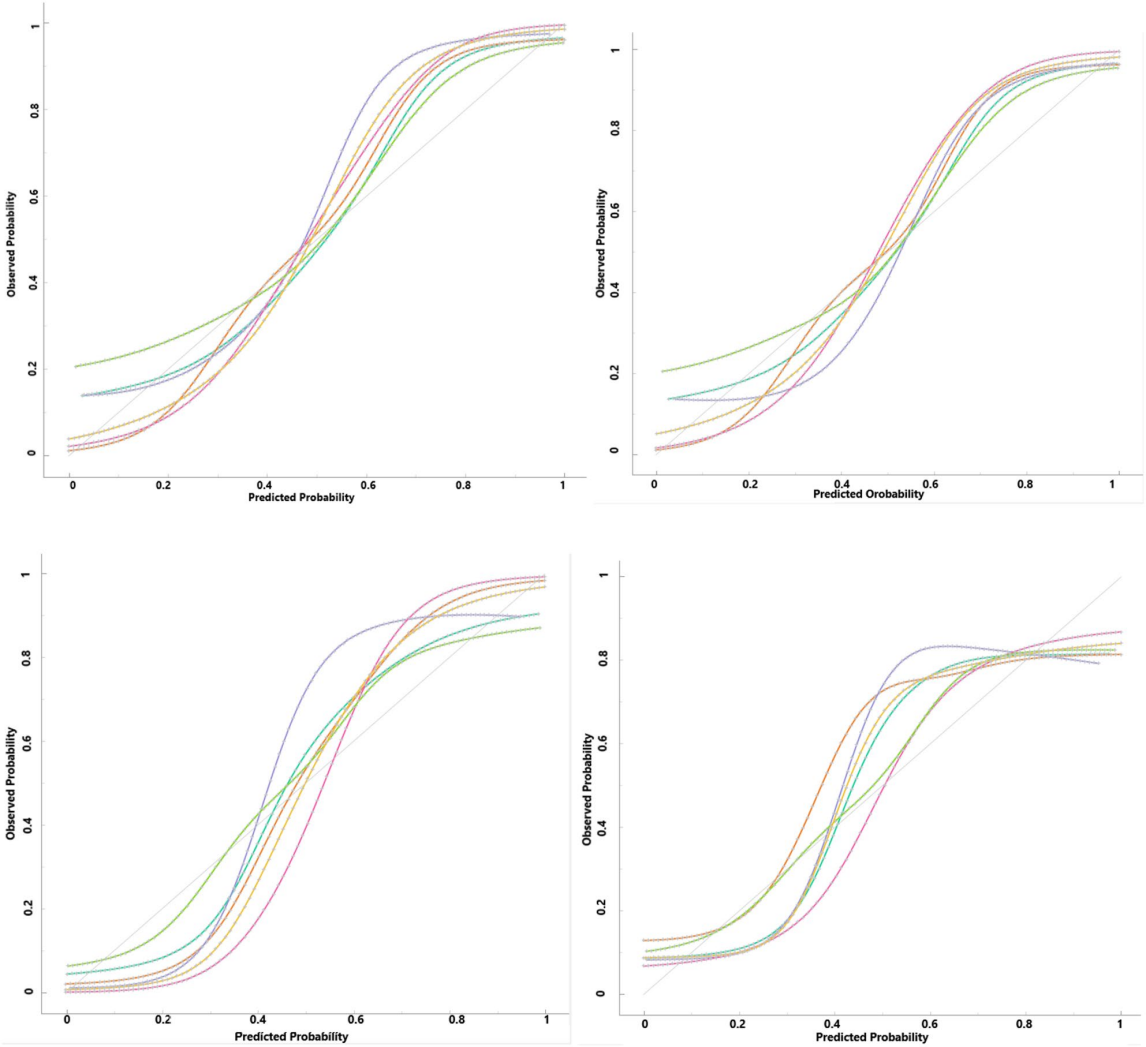
Calibration plots of class probabilities against those predicted by ML algorithms (Top left: Train original data, Top right: Test original data, Down left: Train balanced data, Down right: Test balanced data).

Also, Fig. 5 was presented to compare a model's predicted probability of an event to the empirical probability. If the predicted probability was near to the class membership probability, the model will be well-calibrated. In other world, the closer models curves is to the perfect calibrated curve (dotted line), the better calibrated is obtained. The curves in the figure seem to be the expected S-shaped in all ML-based methods.

**Figure 6:**
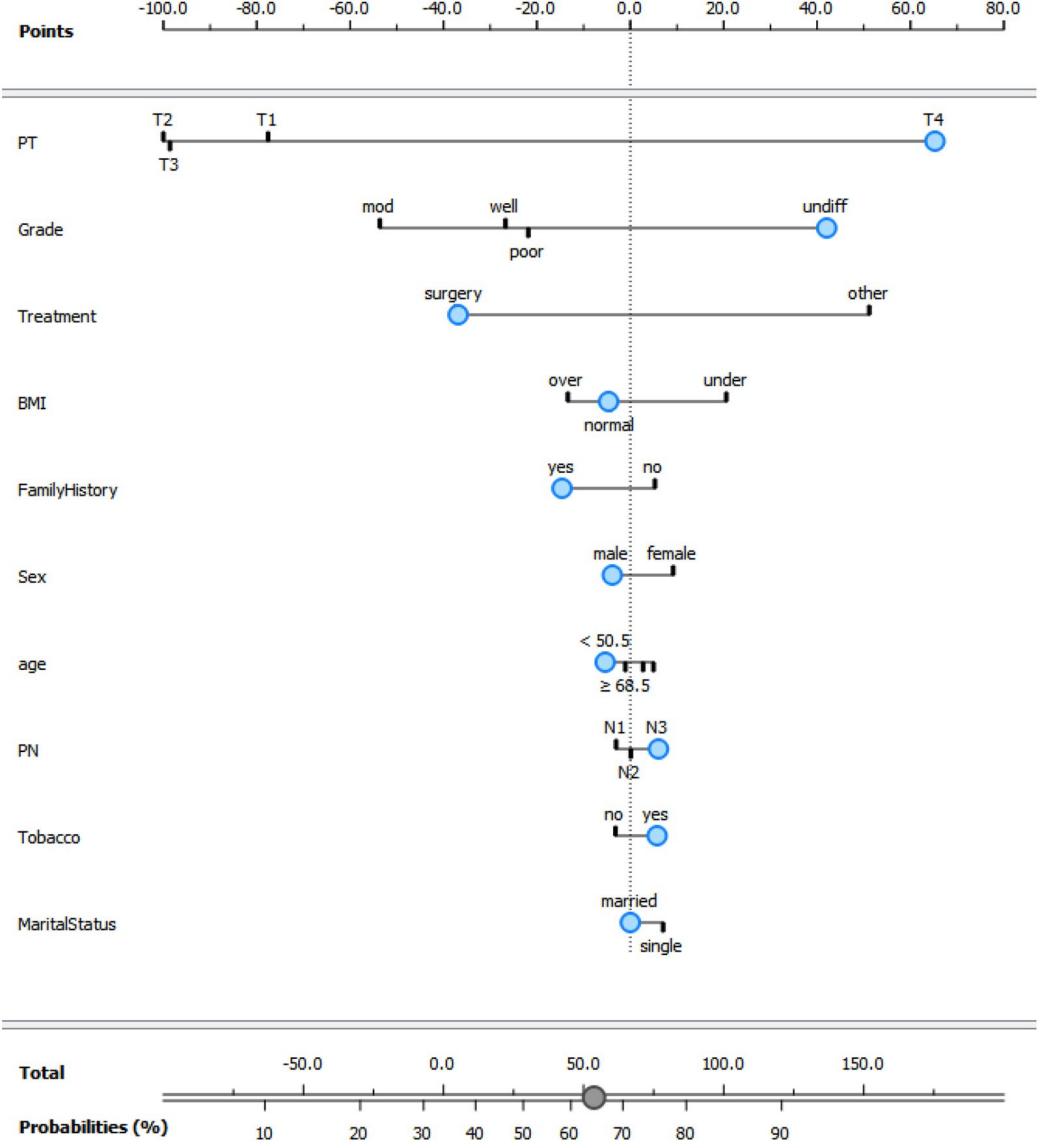
Nomogram for visualization of NB model for predicting metastasis in GC patients.

Then, a nomogram for visualization of NB model has been presented in the Fig. 6. This nomogram can be used to calculate the predicted outcome. In other words, the nomogram represents a graphical model to calculate the individual probability for metastais after entering the risk factors information for a GC patient. After entering the information, the total point score of all variables is summed and the probability of metastasis is determined. The influence of each predictor is determined by the points on the horizontal line at the top of chart. By adding these points, which are associated with the predictors, probability of metastasis will be reach on the response probability horizontal line at the bottom of the chart. The total point will correspond to this probability. For example the blue dots in the nemogram present a patient who has the following characteristics: male (− 5 points), tumor size of T4 (65 points), grade undifferated (45 points), surgery treatment (− 35 points), normal BMI (− 5 points), positive family history (− 15 points), age lower than 50.5 year (− 5 points), number of involved lymph node of N3 (5 points), user tobacco (5 points) and marrid status (0 points); the calculation example shows the total score is approximately 55, which is correspond to the probability of metastasis 65%.

**Figure 7:**
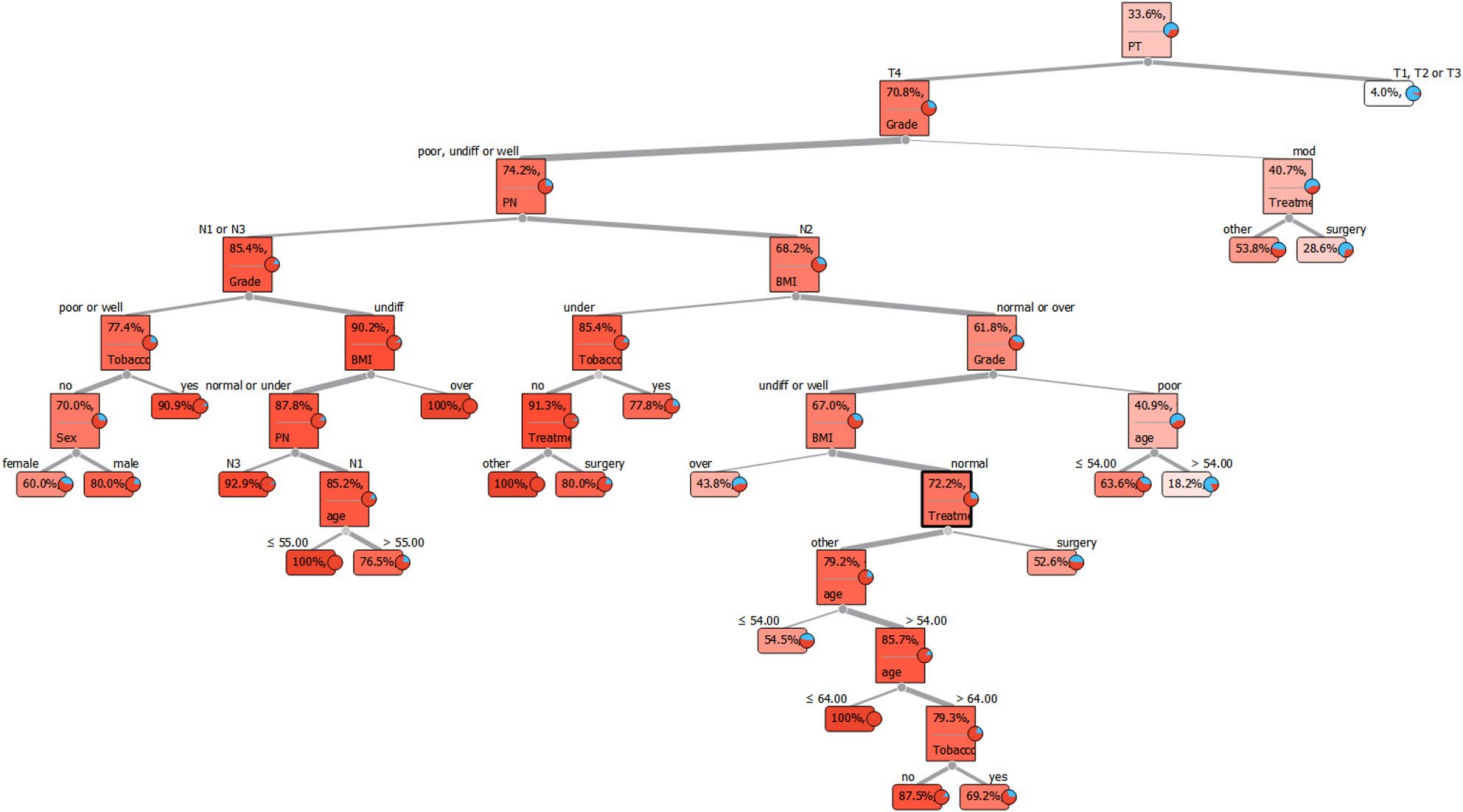
Visualization of decision tree model; The first part is the root node of the tree. To predict the outcome, start from the root node (PT variable) and then go to the next intermediate nodes and the edges show which subsets are visited. Once one reach the final subsets (named leaf node), the predicted outcome is assessed by leaf node.

Finally, a visualization of DT has been illustrated in Fig. 7. To predict the outcome, start from the root node and then go to the next intermediate nodes and the edges show which subsets are looked at. Once one reach the final subsets (named leaf node), the predicted outcome is assesed by leaf node. For example, to predict a person's metastasis status, we first check the PT variable based on the root node. If the PT status is T1, T2 or T3, metastasis will occur only in 4% of cases. When the person's PT status is T4, the grade should be checked according to the next intermediate nodes. If the grade is moderate, treatment will be checked in the next step. At this stage, leaf nodes show whether a person receives surgery treatment, metastasis is predicted in 28.6% of the cases, but if a patient receives other treatments, the probability of metastasis happens in 53.8% of the cases.

Table 3 demonstrated that the most considerable variable was the size of tumor. Then age, grade of tumor, number of lymph nodes and type of treatment played indispensabale roles in predicting metastasis.

**Table 3:** Important variable to predict metastasis in gastric cancer patients.

In order of importance in variables	Gini
Size of tumor	.015
Age	.004
Grade of tumor	.002
The number of involved lymph nodes	.002
Type of treatment	.002
BMI	.001
Marital status	.001
Smoking	.001

## Discussion

The ML-based methods were carried out to predict the metastasis in patients with GC in the study.

Some criteria such as F1 score, sensitivity, specificity, precision, AUC and PR-AUC can be extracted from ROC curve analysis to assess the ML-based models performance. Among those models, SVM and NN can be better predictive model although all those algorithms had the high AUC and sensitivity.

Some ML methods have been proposed in patients with GC until now. Some of them have used ML techniques to study the relationship between lncRNAs and complex diseases in Gene datasets, while some others applied ML strategies, including SVM, RF, NN, GBM and deep learning to predict metastasis situation^23–26^.

A study assesses the performances of seven different ML methods, such as LR, SVM, RF, LASSO, sparse neural network (sNNR), Extreme gradient boosting (XGboost) and stochastic gradient boosting (SGB) to predict GC risk after H. pylori eradication^27^. Based on period of eradication therapy, the data was divided into two train and test datasets. The AUC was obtained to calculate the model performance. The results of the study revealed that the XGboost was considered as the most successful among all seven models; however, the SVM was had the lowest sensitivity, specificity and AUC, which was inconsistent with our finding that SVM was regarded as the best ML algorithm. In their study, age, smoking, drinking, comorbities, need of Helicobacter pylory retreatment, medications were significant in both high and low- risk patients. Some of variables such as drinking, comorbities, need of Helicobacter pylory retreatment, medications were not taken in our study. On the contrary, size of tumor and age were as essential variables, which was compatible in our study that age in RF serves as significant variable.

Akcay et al. (2020) investigated the overall survival (OS) and recurrence patterns by ML algorithms in patients with radiation therapy^28^. The goal of the study was to fit the ML approaches, including LR, XGBoost, SVM, RF, multilayer perceptron (MLP) and Gaussian Naive Bayes (GNB) in the assessment of the OS, distant metastasis (DM), and peritoneal recurrence (PR) prediction. The best performance models in the prediction of OS, distant metastases, and peritoneal metastases were discoverd to be GNB, XGBoost and RF, respectively. Also, in their study GNB was considered as the better model to evaluate the OS, but all ML-based approaches had ideal performances in our study and it seems that SVM act as a better model among 6 methods. The almost identical AUC of this survey was consistent with our study on GC patients.

A clinical study applied ML approaches to predict lymph node metastasis in GC patients^29^. The result of the study showed that tumor size, grade of tumor, depth of tumor and age were significant (*P* < 0.001). The essential factors in the study were consistent with our study that age, tumor size and grade were significant. Also, their results presented that NN model had maximum and gradient boosting machine (GBM) method had the minimum sensitivity and specificity among seven ML-based algorithms, respectively. The result of this study was consistent with our study, that NN model was regarded as the successful model among 6 methods.

Zhou et al. established the prediction of metastasis in GC patients using 5 techniques of MLs^16^. Those methods were LR, RF, DT, GBM and Light Gradient Boosting. The most and the least AUC were related to gbm and DT, respectively. The minimum AUC in the study was compatible with our study (AUC = 0.75 in test dataset). Furthermore, the prime variables were tumor size, pathological type and depth of invasion in the study; nevertheless, tumor size, age, grade of tumor, the number of involved lymph node, treatment type, were significant in our study. Also, the precision of RF model is a little better than other models.

As a limitation of the present study, we have to state the small size of the dataset (733 patients): a larger dataset would have let us to extract more valid results. Additional factors about the th GC patients (race, drinking, depth of tumor, stage of tumor, primary site, etc.) and their activity and nutrition factors would have been useful to challenge additional risk factors for GC. Also, if another dataset with the same features from a different geographical region had been accessible, we would have applied it as a validation cohort to establish our outputs.

## Conclusion

Among 6 presented models in our study, SVM was considered as top approach. Then, NN and RF can be better as better ML-based algorithms among 6 methods. Also, tumor size, age, grade of tumor, the number of involved lymph node, treatment type, BMI, marital status and history of smoking play a crucial role in patients with GC in RF model.

## Supplementary Information

Supplementary Information.

## Data Availability

The datasets used and analyzed during the current study are available from the corresponding author on reasonable request.

## Abbreviations

AUC: Area under the curve
DT: Decision tree
GC: Gastric cancer
LR: Logistic regression
ML: Machine learning
NB: Naïve Bayes
NN: Neural network
RF: Random forest
ROC: Receiver operating characteristic curve
SVM: Support vector machine
