# Author Correction: Predicting metastasis in gastric cancer patients: machine learning-based approaches

**DOI:** 10.1038/s41598-023-34810-8

**Published:** 2023-05-24

**Authors:** Atefeh Talebi, Carlos A. Celis-Morales, Nasrin Borumandnia, Somayeh Abbasi, Mohamad Amin Pourhoseingholi, Abolfazl Akbari, Javad Yousefi

**Affiliations:** 1 Colorectal Research Center, Iran University of Medical Sciences, Tehran, Iran; 2 British Heart Foundation Cardiovascular Research Centre, University of Glasgow, Glasgow, UK; 3 Institute of Cardiovascular and Medical Sciences, University of Glasgow, Glasgow, UK; 4 Urology and Nephrology Research Center, Shahid Beheshti University of Medical Sciences, Tehran, Iran; 5 Department of Mathematics, Isfahan (Khorasgan) Branch, Islamic Azad University, Isfahan, Iran; 6 Gastroenterology and Liver Diseases Research Center, Research Institute for Gastroenterology and Liver Diseases, Shahid Beheshti University of Medical Sciences, Tehran, Iran; 7 Colorectal Research Center, Iran University of Medical Sciences, Tehran, Iran; 8 Department of Internal Medicine, School of Medicine, Iran University of Medical Sciences, Tehran, Iran

Correction to: *Scientific Reports* 10.1038/s41598-023-31272-w, published online 13 March 2023

The original version of this Article contained errors in Affiliations 1 and 8, which were incorrectly given as ‘Colorectal Research Center, Iran University of Medical Center, Tehran, Iran’ and as ‘Department of Internal Medicine, Iran University of Medical Sciences, Tehran, Iran’, respectively. The correct affiliations are listed below.

^1^Colorectal Research Center, Iran University of Medical Sciences, Tehran, Iran.

^8^Department of Internal Medicine, School of Medicine, Iran University of Medical Sciences, Tehran, Iran.

The original Article has been corrected.

