# Supplementary material for: Predicting metastasis in gastric cancer patients: machine learning-based approaches
Source: Sci Rep. 2023 Mar 13;13:4163. doi: 10.1038/s41598-023-31272-w (PMC10011363; doi:10.1038/s41598-023-31272-w)
Supplement: Supplementary file 1 — Supplementary Information. [file 41598_2023_31272_MOESM1_ESM.docx]

**SUPPLEMENTARY MATERIAL**

**Supplemental Figures**


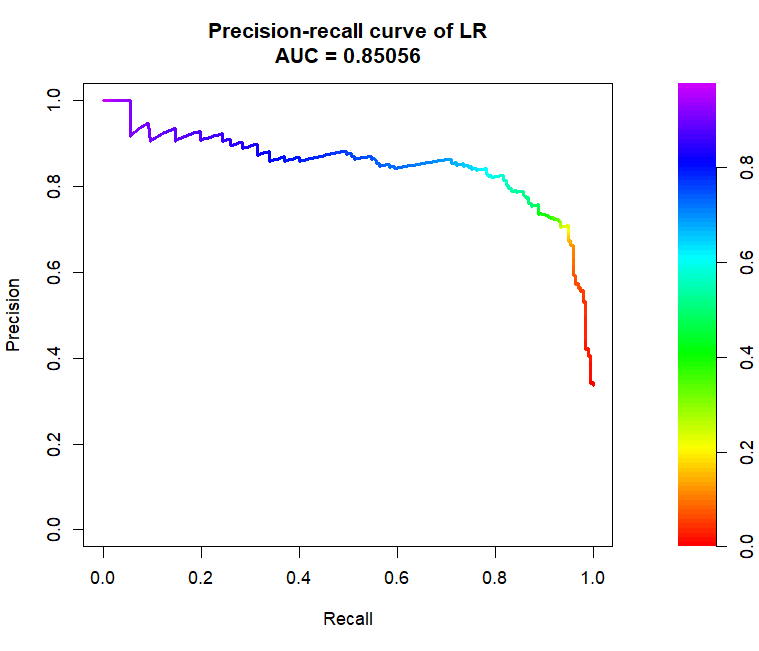

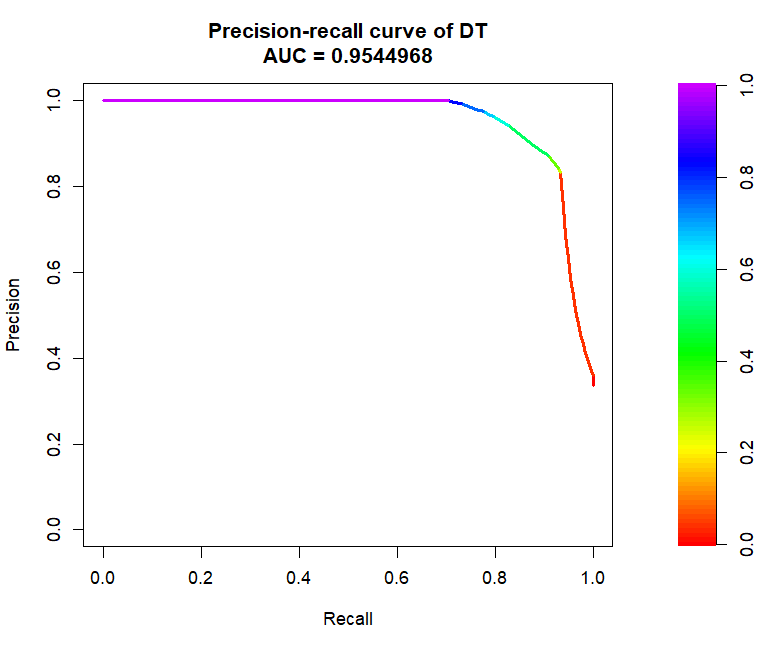


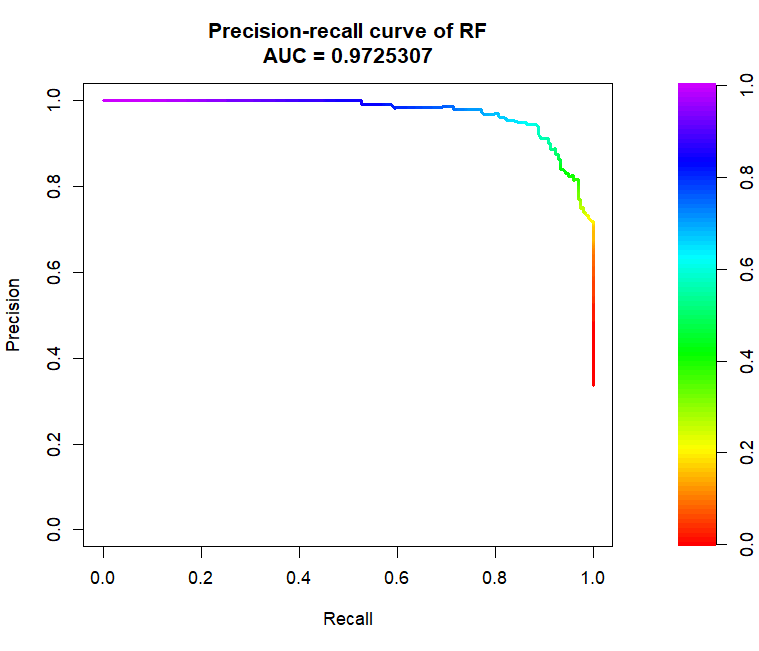

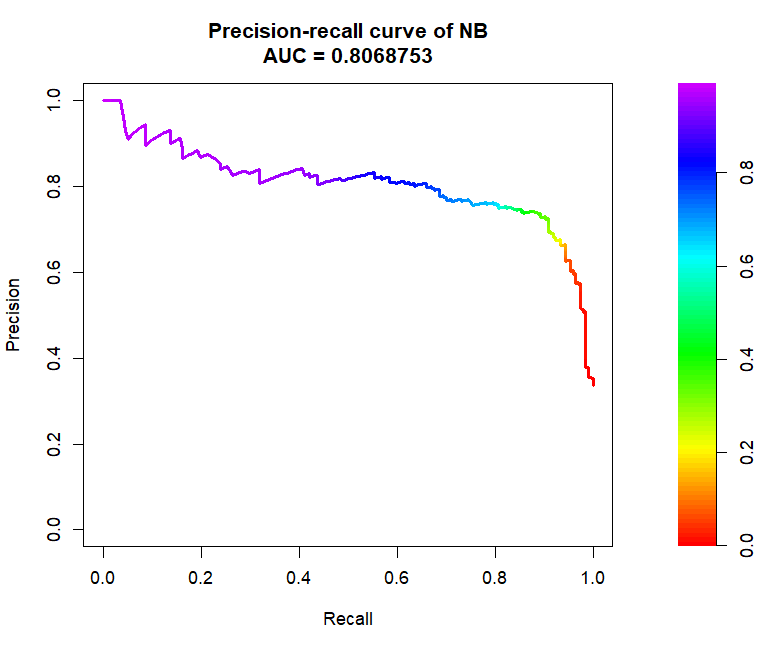

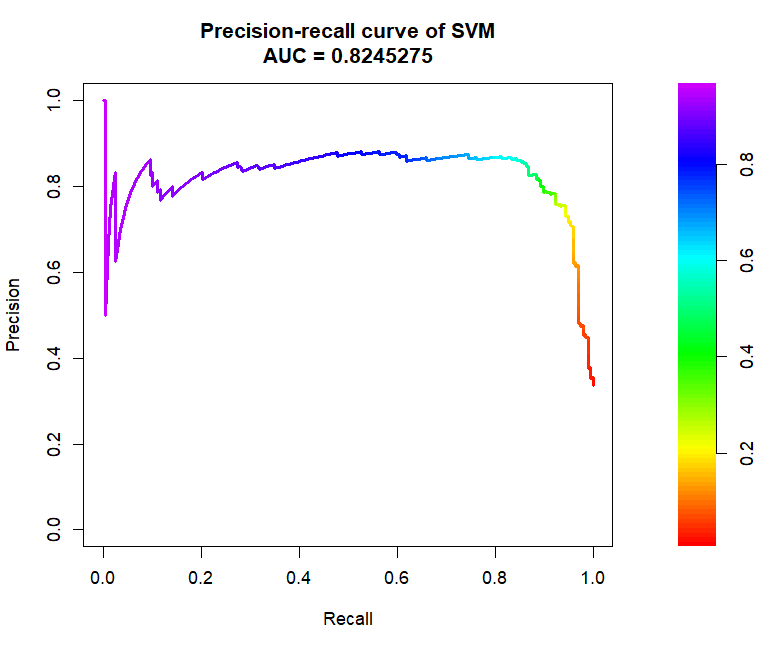

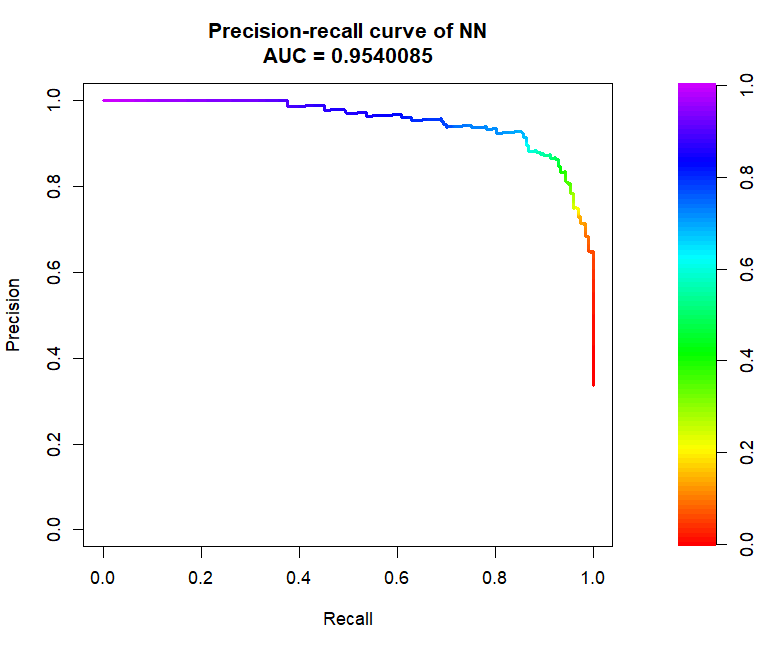


**Figure 1.** Precision-recall curve and its AUC in six ML algorithms of original train dataset of GC patients


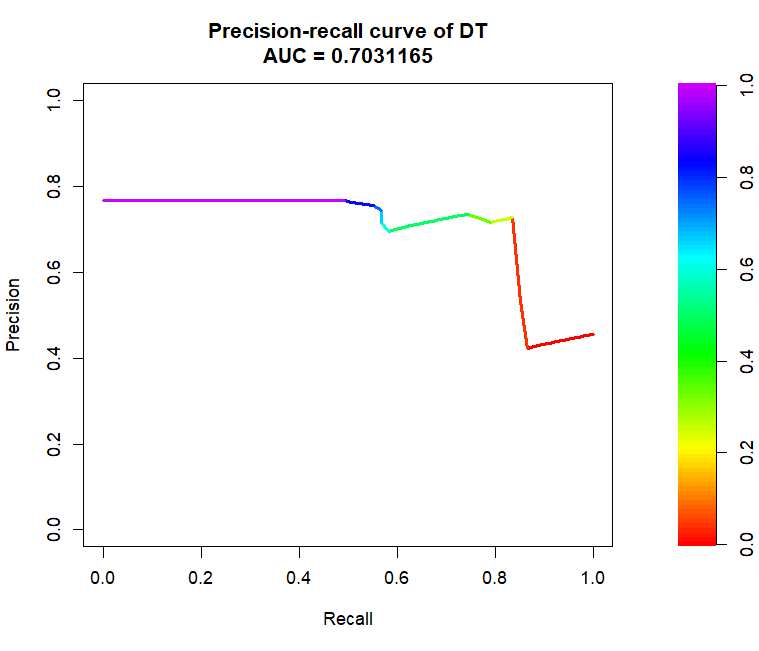

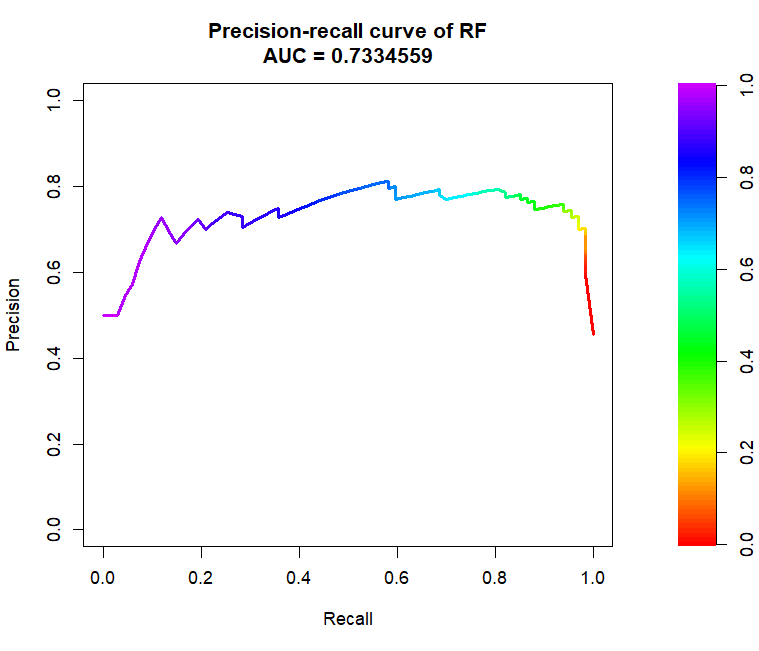

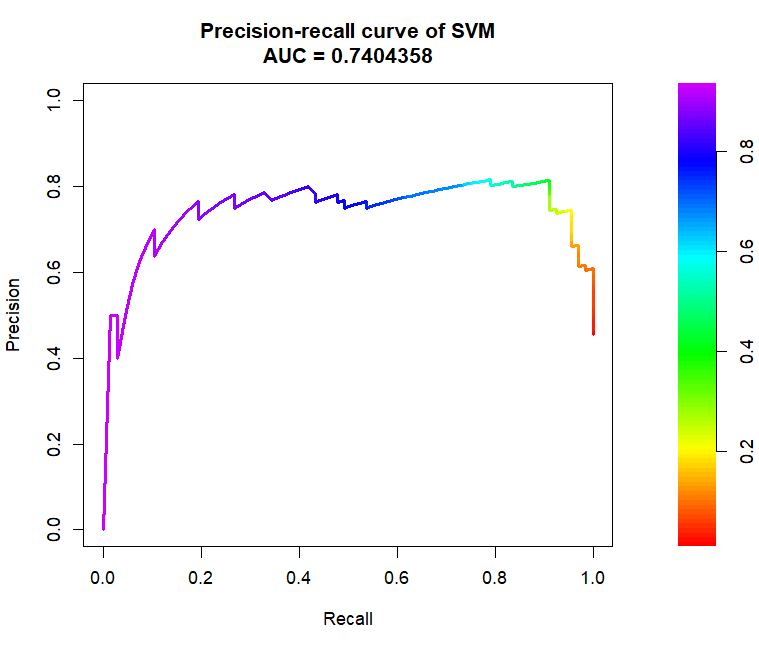

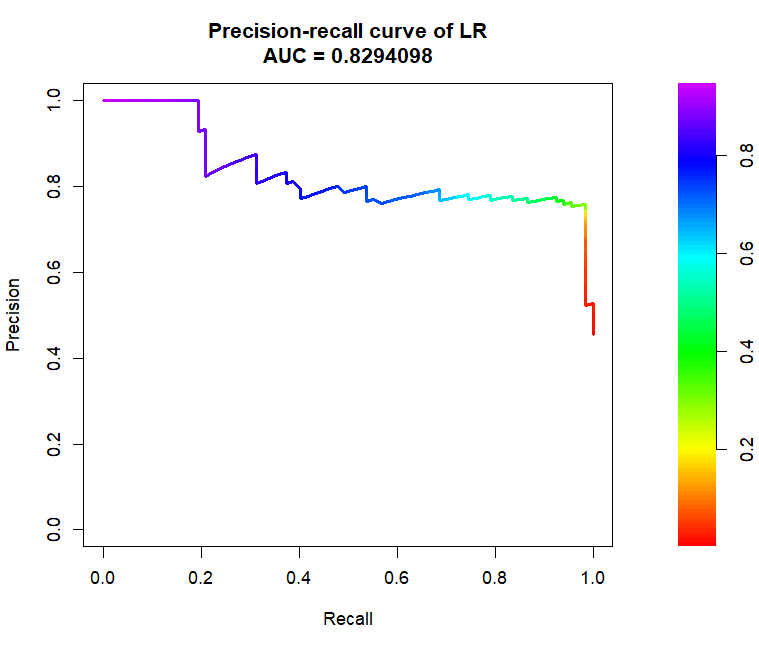


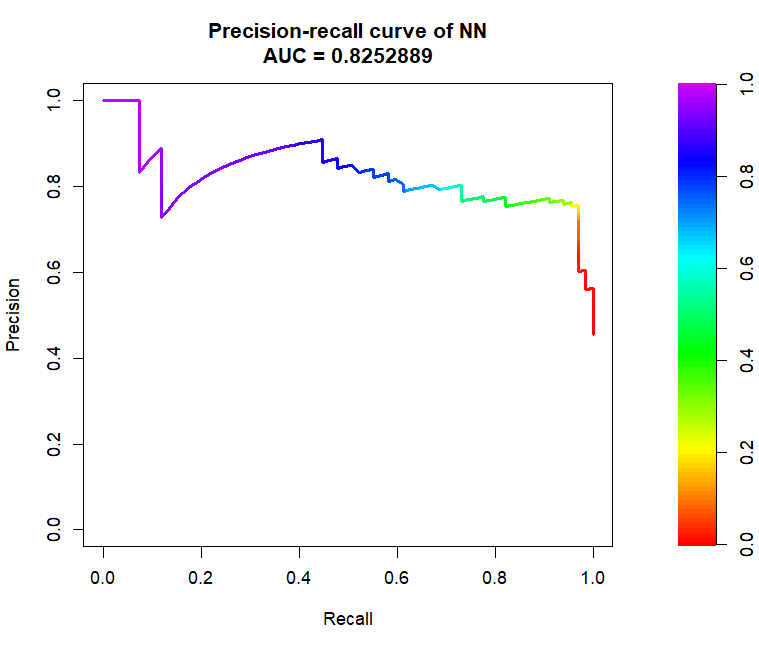


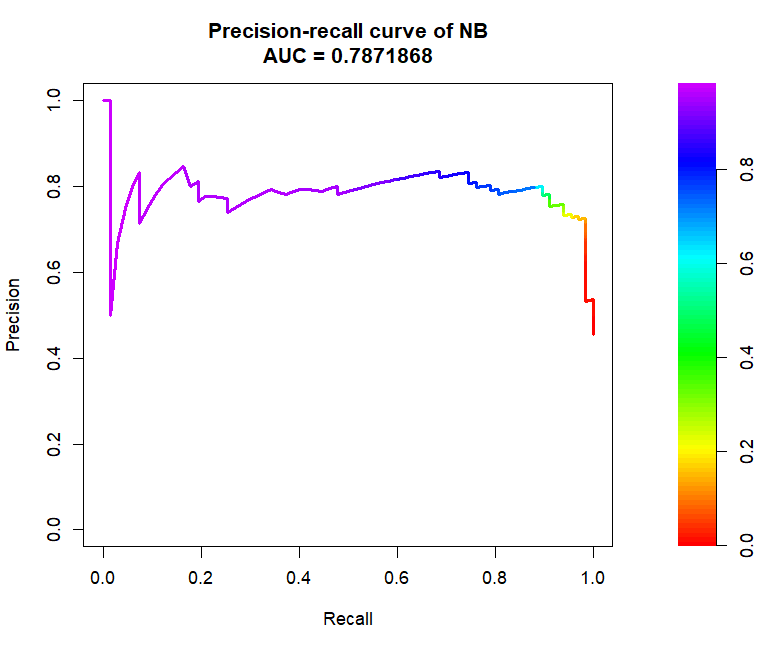


**Figure 2.** Precision-recall curve and its AUC in six ML algorithms of original test dataset of GC patients
